# 

**Published:** 1904-12

**Authors:** 


*THE BRISTOL MEDICO-CHIRURGICAL SOCIETY.*

CONTENTS.

| | PAGE |
|---|---|
| **Problems in Psychology.** By REGINALD EAGER, M.D.Lond. ... ... ... ... ... | 289 |
| **The Long Fox Lecture.** By JOHN BEDDOE, M.D., LL.D., F.R.S. ... ... ... ... ... | 303 |
| **On Torsion of the Spermatic Cord, with the Report of a Recent Case.** By J. LACY FIRTH, M.S.Lond., F.R.C.S . | 320 |
| **Case of Mesenteric Cyst.** By A. W. PRICHARD, M.R.C.S. ... ... ... ... ... | 332 |
| **Progress of the Medical Sciences:** | |
| **Medicine.** J. MICHELL CLARKE, M.D. Cantab.... ... ... ... ... ... ... | 334 |
| **Progress of Medical Sciences:** | |
| **Surgery.** JAMES SWAIN, M.D., M.S. Lond. | 339 |
| **Dermatology.** W. KENNETH WILLS, M.B., B.C. Cantab. ... ... ... ... ... | 342 |
| **Reviews of Books** ... ... ... ... ... ... | 347 |
| **Editorial Notes** ... ... ... ... ... ... | 362 |
| **Notes on Preparations for the Sick** ... | 366 |
| **The Library of the Bristol Medico-Chirurgical Society** ... ... ... ... | 374 |
| **Meetings of Societies** ... ... ... ... ... | 377 |
| **Local Medical Notes** ... ... ... ... ... | 382 |

